# Crystal structure of (+)-methyl (*E*)-3-[(2*S*,4*S*,5*R*)-2-amino-5-hy­droxy­meth­yl-2-tri­chloro­methyl-1,3-dioxolan-4-yl]-2-methyl­prop-2-enoate

**DOI:** 10.1107/S2056989016002474

**Published:** 2016-02-17

**Authors:** Takeshi Oishi, Daichi Yasushima, Kihiro Yuasa, Takaaki Sato, Noritaka Chida

**Affiliations:** a School of Medicine, Keio University, Hiyoshi 4-1-1, Kohoku-ku, Yokohama 223-8521, Japan; b Department of Applied Chemistry, Faculty of Science and Technology, Keio University, Hiyoshi 3-14-1, Kohoku-ku, Yokohama 223-8522, Japan

**Keywords:** crystal structure, ortho­amide, dioxolane, hydrogen bonding, hy­droxy group, amino group, disorder

## Abstract

In the title compound, C_10_H_14_Cl_3_NO_5_, the five-membered dioxolane ring adopts an envelope conformation. The C atom at the flap, which is bonded to the hy­droxy­methyl substituent, deviates from the mean plane of other ring atoms by 0.357 (5) Å. There are two intra­molecular hydrogen bonds (O—H⋯N and N—H⋯O) between the hy­droxy and amino groups, so that O- and N-bound H atoms involved in these hydrogen bonds are each disordered with equal occupancies of 0.50. The methyl 2-methyl­prop-2-enoate substituent also shows a disordered structure over two sets of sites with refined occupancies of 0.482 (5) and 0.518 (5). In the crystal, mol­ecules are connected into a dimer by an O—H⋯O hydrogen bond. The dimers are further linked by N—H⋯O, C—H⋯N and C—H⋯O inter­actions, extending a sheet structure parallel to (01).

## Abstract 2

In the title compound, intra­molecular hydrogen bonding between the hy­droxy and amino groups forms an *S*(7) graph-set motif. In the crystal, an inter­molecular O—H⋯O hydrogen bond connects mol­ecules into a dimer. The dimers are further linked into a sheet structure.

## Chemical context   

The 3,3-sigmatropic rearrangement of an allylic tri­chloro­acetimidate (Overman rearrangement; Overman, 1974 ▸, 1976 ▸) is one of the most important reactions in organic chemistry. It has been utilized as a quite powerful tool to introduce the nitro­gen functional group because this imidate is easily available from an allylic alcohol with tri­chloro­aceto­nitrile (Cl_3_CC N). In the case of a diol, a cyclic ortho­amide (2-amino-2-tri­chloro­methyl-1,3-dioxolane) may be afforded by controlling the reaction conditions though bis-imidates are usually produced. We have explored the rearrangement of the cyclic ortho­amide prepared from a contiguous diol or triol, and have developed a novel strategy for the total synthesis of certain natural products (Nakayama *et al.*, 2013 ▸). As part of our ongoing studies in this area, we now describe the synthesis and structure of the title compound.
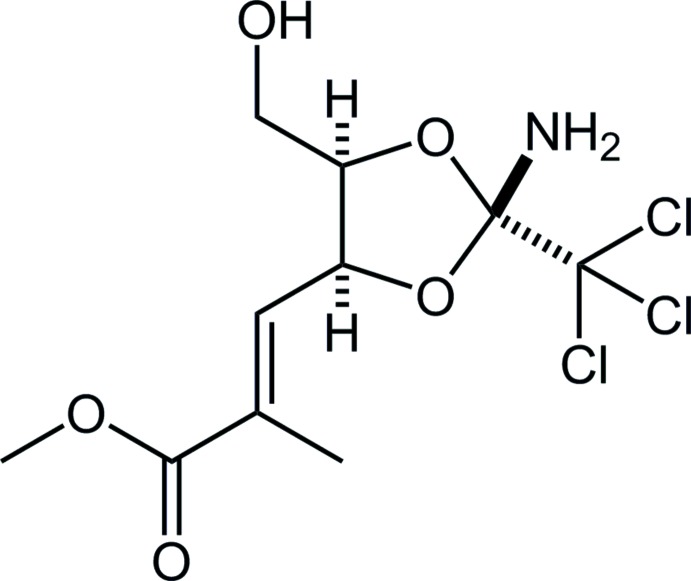


## Structural commentary   

**Figure 1:**
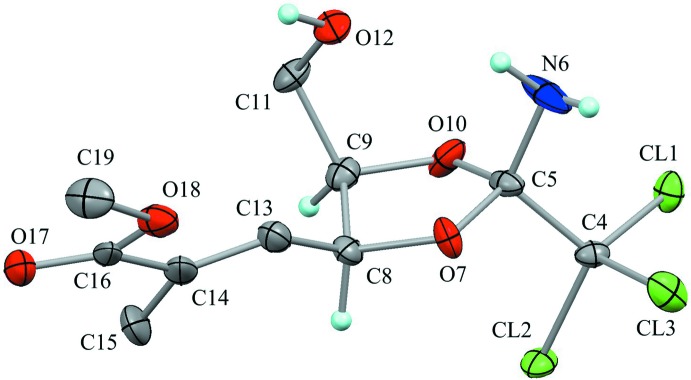
The mol­ecular structure of the title compound, showing the atom labelling. Displacement ellipsoids are drawn at the 50% probability levels. Only H atoms connected to O, N and chiral C atoms are shown for clarity. Other possible positions of disordered atoms have been omitted.

**Figure 2:**
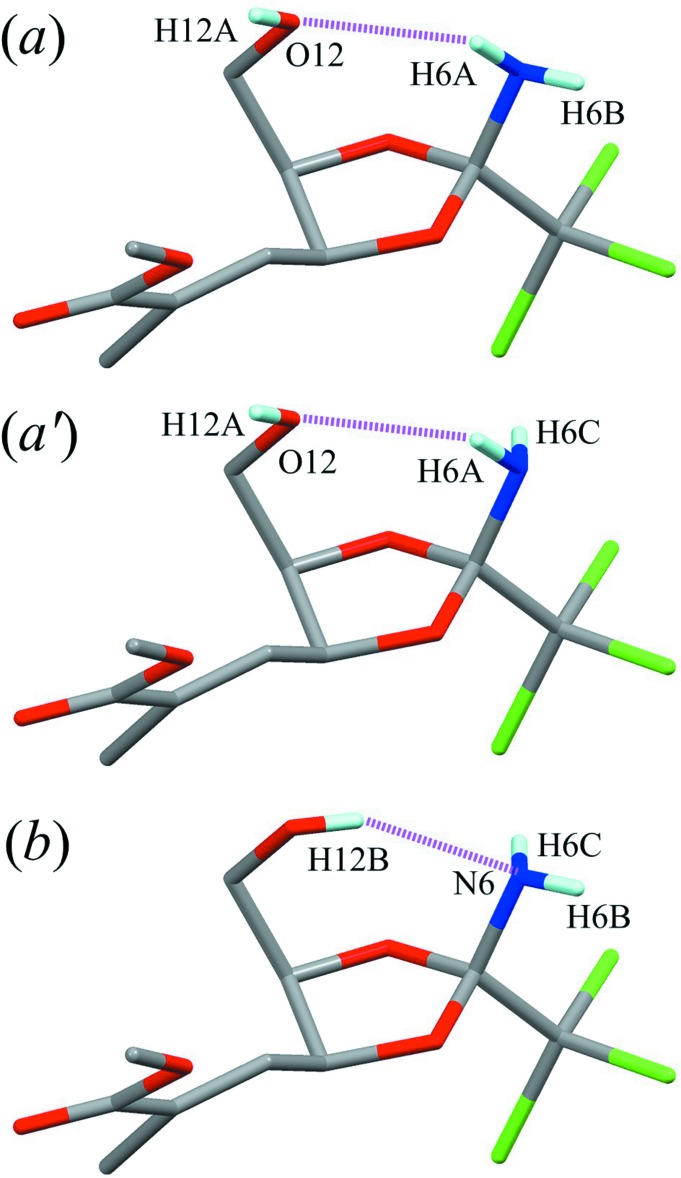
Three possible combinations of the hy­droxy and amino H atoms, the probabilities being (*a*) 25%, (*a*′) 25% and (*b*) 50%. Purple dotted lines indicate the intra­molecular N—H⋯O and O—H⋯N hydrogen bonds. Other H atoms have been omitted for clarity.

The mol­ecular structure of the title compound is shown in Fig. 1 ▸. The dioxolane ring (C5/O7/C8/C9/O10) adopts an envelope form with puckering parameters of *Q*(2) = 0.223 (3) Å and *φ*(2) = 111.7 (8)°. Atom C9 deviates from the mean plane of the other four atoms by 0.357 (5) Å. The hy­droxy H atom has two possible positions and the amino H atoms have three possible positions, generating two types of intra­molecular hydrogen bonds with an *S*(7) graph-set motif between the hy­droxy and amino groups, N6—H6*A*⋯O12 and O12—H12*B*⋯N6 (Table 1 ▸ and Fig. 2 ▸). The occupation factor of atoms H12*A*, H12*B* and H6*A* is 0.5, while that of atoms H6*B* and H6*C* is 0.75. The unsaturated ester substituent (C15/C14/C16/O17/O18/C19) is disordered over two orientations with refined occupancies of 0.482 (5) and 0.518 (5).

## Supra­molecular features   

**Figure 3:**
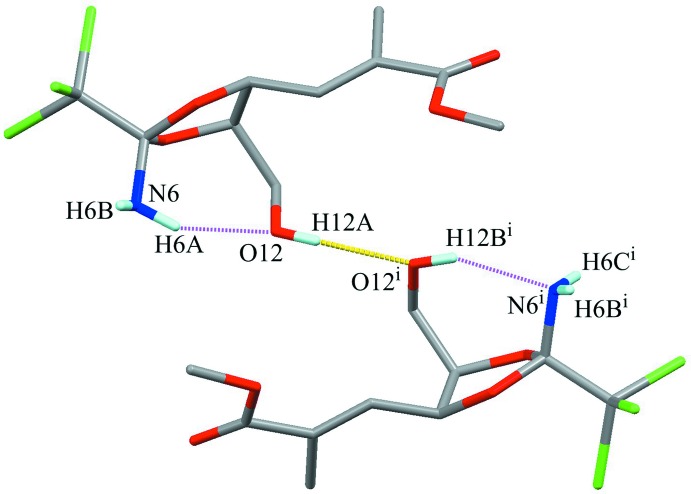
A pair of mol­ecules showing a correlation between the intra- and inter­molecular hydrogen bonds. A yellow dashed line indicates the inter­molecular O—H⋯O hydrogen bond. Purple dotted lines indicate the intra­molecular N—H⋯O and O—H⋯N hydrogen bonds. Only the H atoms of the hy­droxy and amino groups are shown for clarity. The other possible position of N-bound H atoms due to the disorder are omitted. [Symmetry code: (i) −*x* + 1, *y*, −*z* + 1.]

**Figure 4:**
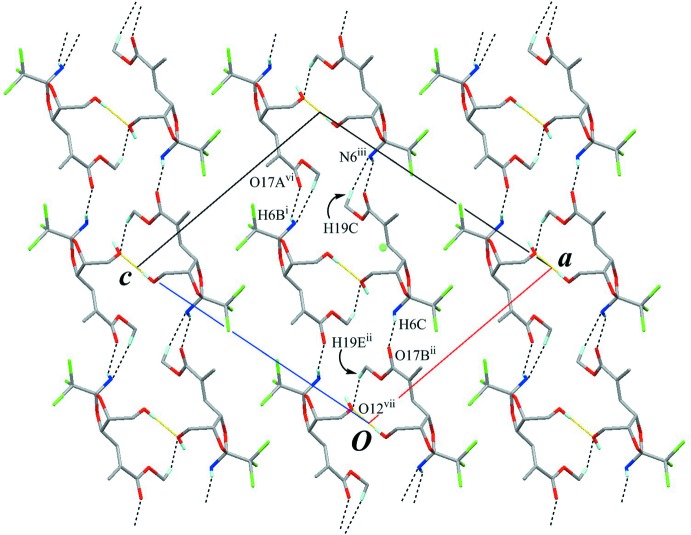
A packing diagram viewed down to the *b* axis. Yellow lines indicate the inter­molecular O—H⋯O hydrogen bonds, generating the dimers. Black dashed lines indicate the inter­molecular N—H⋯O, C—H⋯N and C—H⋯O inter­actions. Only H atoms involved in hydrogen bonds are shown for clarity. [Symmetry codes: (i) −*x* + 1, *y*, −*z* + 1; (ii) *x* − 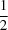, *y* − 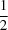, *z* − 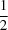; (iii) *x* + 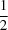, *y* + 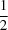, *z* + 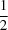; (vi) −*x* + 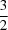, *y* + 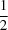, −*z* + 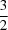; (vii) −*x* + 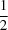, *y* + 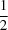, −*z* + 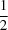.]

The crystal packing is stabilized by O—H⋯O hydrogen bonding (O12—H12*A*⋯O12^i^; Table 1 ▸), connecting mol­ecules related by a twofold rotation axis into a dimer. As the result of this inter­molecular linkage, the intra­molecular hydrogen-bonding pattern is restricted, as shown in Fig. 3 ▸. The dimers are further linked by weak N—H⋯O, C—H⋯N and C—H⋯O inter­actions (N6—H6*C*⋯O17*B* ^ii^ and N6—H6*B*⋯O17*A* ^iv^, C19*A*—H19*C*⋯N6^iii^ and C19*B*—H19*E*⋯O12^v^; Table 1 ▸, Figs. 4 ▸ and 5 ▸) to form a sheet structure parallel to (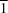
01).

## Database survey   

**Figure 6:**
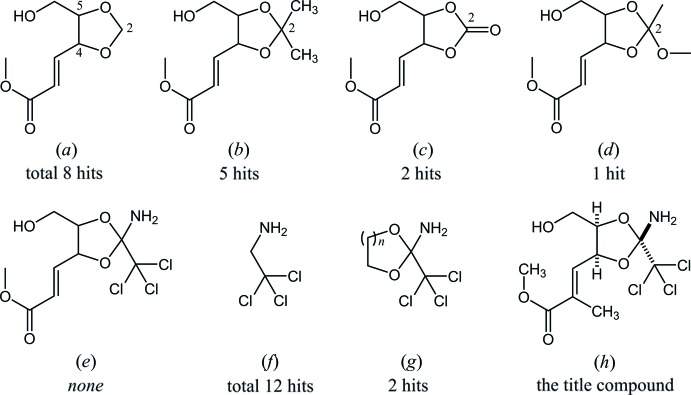
The core structures for database survey; (*a*) 5-hy­droxy­methyl-4-(prop-2-enoate-3-yl) substituted 1,3-dioxolane, and its (*b*) 2,2-dimethyl, (*c*) 2-oxo, (*d*) 2-alk­oxy-2-alkyl and (*e*) 2-amino-2-tri­chloro­methyl derivatives, (*f*) 2,2,2-tri­chloro­ethan-1-amine, and its derivatives (*g*) 2-amino-2-tri­chloro­methyl-1,3-dioxolane (*n* = 1) or -1,3-dioxane (*n* = 2); and (*h*) the structure of the title compound.

In the Cambridge Structural Database (CSD, Version 5.36, November 2014; Groom & Allen, 2014 ▸), eight structures possessing a 1,3-dioxolane core with 4-(prop-2-enoate-3-yl) and 5-hy­droxy­methyl substituents, (*a*), are registered (Fig. 6 ▸). These include its 2,2-dimethyl, (*b*), 2-oxo, (*c*) and 2-alk­oxy-2-alkyl (orthoester) derivative, (*d*), but its 2-amino-2-tri­chloro­methyl derivative, (*e*), which is related to the title compound, (*h*), has not been reported.

On the other hand, a search in CSD for a 2,2,2-tri­chloro­ethan-1-amine skeleton, (*f*), gave 12 entries. These include two structures (LIBHIO: Rondot *et al.*, 2007 ▸; WEKWOY: Haeckel *et al.*, 1994 ▸) with a 2-amino-2-tri­chloro­methyl-1,3-dioxolane or -1,3-dioxane core, (*g*). N-bound hydrogen atoms in the structure of LIBHIO were refined as having an *sp* ^3^ configuration and tilted towards chlorine atoms, whereas those in other 11 structures were refined assuming an *sp* ^2^ configuration of the N atom.

## Synthesis and crystallization   

The title compound was derived from d-erythrose, which was prepared according to the reported procedure (Storz *et al.*, 1999 ▸) from d-glucose (Yasushima *et al.*, 2016 ▸). Purification was carried out by silica gel column chromatography, and colourless crystals were obtained from a toluene solution by slow evaporation at ambient temperature. M.p. 365–366 K. [*α*]^20^_D_ + 33.8 (*c* 0.32, CHCl_3_).

## Refinement   

Crystal data, data collection and structure refinement details are summarized in Table 2 ▸. The unsaturated ester group is disordered; the atoms C15/C16/O17/O18/C19 were split into two sets of positions *A* and *B* with their geometries restrained, and the refined occupancies being 0.482 (5) and 0.518 (5), respectively.

C-bound H atoms were positioned geometrically with C—H = 0.95–1.00 Å, and constrained to ride on their parent atoms with *U*_iso_(H) = 1.2*U*_eq_(C) or 1.5*U*_eq_(methyl C). The O-bound hydrogen atom has two possible positions. They were refined isotropically with *U*_iso_(H) = 1.5*U*_eq_(O) and the O—H distances restrained. The N-bound hydrogen atoms have three possible positions. They were refined isotropically with *U*_iso_(H) = 1.2*U*_eq_(N) and the N—H and H⋯H distances restrained. The site-occupation factors of the disordered H atoms of the hy­droxy group (H12*A* and H12*B*) and one of the amino group (H6*A*) were uniquely assigned to 0.5 each based on the two possible patterns of the hydrogen-bonding linkages related by the twofold axis. The occupation factors of the other N-bound H6*B* and H6*C* atoms were assumed to be 0.75 each from a difference map.

## Supplementary Material

Crystal structure: contains datablock(s) global, I. DOI: 10.1107/S2056989016002474/is5442sup1.cif

Structure factors: contains datablock(s) I. DOI: 10.1107/S2056989016002474/is5442Isup2.hkl

Supporting information file. DOI: 10.1107/S2056989016002474/is5442Isup3.cml

CCDC reference: 1452471

Additional supporting information:  crystallographic information; 3D view; checkCIF report

## Figures and Tables

**Figure 5:**
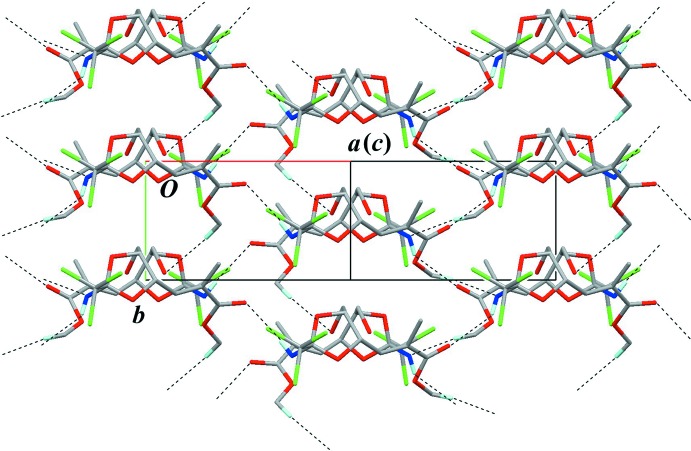
A partial packing diagram viewed along [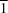
01], showing hydrogen bonding in the sheet structure. Overlapped mol­ecules indicate the dimer. Black dashed lines indicate the inter­molecular N—H⋯O, C—H⋯N and C—H⋯O inter­actions. Only H atoms involved in hydrogen bonds are shown for clarity.

**Table 1:** Hydrogen-bond geometry (Å, °)

*D*—H⋯*A*	*D*—H	H⋯*A*	*D*⋯*A*	*D*—H⋯*A*
O12—H12*B*⋯N6	0.82 (3)	2.22 (4)	2.986 (4)	155 (7)
N6—H6*A*⋯O12	0.87 (3)	2.31 (5)	2.986 (4)	135 (6)
O12—H12*A*⋯O12^i^	0.84 (3)	1.98 (4)	2.750 (4)	151 (5)
N6—H6*C*⋯O17*B* ^ii^	0.81 (2)	2.57 (2)	3.369 (7)	169 (3)
C19*A*—H19*C*⋯N6^iii^	0.98	2.59	3.555 (15)	167
N6—H6*B*⋯O17*A* ^iv^	0.85 (2)	2.61 (5)	3.286 (7)	137 (5)
C19*B*—H19*E*⋯O12^v^	0.98	2.62	3.573 (11)	164

Symmetry codes: (i) 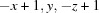; (ii) 
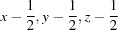
; (iii) 
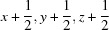
; (iv) 
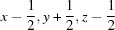
; (v) 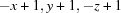.

**Table 2:** Experimental details

Crystal data	
Chemical formula	C_10_H_14_Cl_3_NO_5_
*M*_r_	334.57
Crystal system, space group	Monoclinic, *I*2
Temperature (K)	90
*a*, *b*, *c* (Å)	14.8821 (9), 5.5847 (3), 17.2971 (14)
β (°)	105.847 (2)
*V* (Å^3^)	1382.96 (16)
*Z*	4
Radiation type	Mo *K*α
μ (mm^−1^)	0.68
Crystal size (mm)	0.26 × 0.22 × 0.16

Data collection	
Diffractometer	Bruker D8 Venture
Absorption correction	Multi-scan (*SADABS*; Bruker, 2014 ▸)
*T*_min_, *T*_max_	0.84, 0.90
No. of measured, independent and observed [*I* > 2σ(*I*)] reflections	11488, 2420, 2272
*R*_int_	0.030
(sin θ/λ)_max_ (Å^−1^)	0.595

Refinement	
*R*[*F* ^2^ > 2σ(*F* ^2^)], *wR*(*F* ^2^), *S*	0.023, 0.052, 1.03
No. of reflections	2420
No. of parameters	207
No. of restraints	36
H-atom treatment	H atoms treated by a mixture of independent and constrained refinement
Δρ_max_, Δρ_min_ (e Å^−3^)	0.29, −0.21
Absolute structure	Flack *x* determined using 959 quotients [(*I* ^+^)−(*I* ^−^)]/[(*I* ^+^)+(*I* ^−^)] (Parsons *et al.*, 2013 ▸)
Absolute structure parameter	−0.02 (2)

Computer programs: *APEX2* (Bruker, 2014 ▸), *SAINT* (Bruker, 2014 ▸), *SHELXS2013* (Sheldrick, 2008 ▸), *SHELXL2014* (Sheldrick, 2015 ▸), *Mercury* (Macrae *et al.*, 2006 ▸), *publCIF* (Westrip, 2010 ▸) and *PLATON* (Spek, 2009 ▸).

## supplementary crystallographic information

### Crystal data

**Table:**

C_10_H_14_Cl_3_NO_5_	*D*_x_ = 1.607 Mg m^−^^3^
*M**_r_* = 334.57	Melting point = 366–365 K
Monoclinic, *I*2	Mo *K*α radiation, λ = 0.71073 Å
*a* = 14.8821 (9) Å	Cell parameters from 6489 reflections
*b* = 5.5847 (3) Å	θ = 2.5–24.9°
*c* = 17.2971 (14) Å	µ = 0.68 mm^−^^1^
β = 105.847 (2)°	*T* = 90 K
*V* = 1382.96 (16) Å^3^	Prism, colourless
*Z* = 4	0.26 × 0.22 × 0.16 mm
*F*(000) = 688	

### Data collection

**Table:**

Bruker D8 Venture diffractometer	2420 independent reflections
Radiation source: fine-focus sealed tube	2272 reflections with *I* > 2σ(*I*)
Multilayered confocal mirror monochromator	*R*_int_ = 0.030
Detector resolution: 10.4167 pixels mm^-1^	θ_max_ = 25.0°, θ_min_ = 2.5°
φ and ω scans	*h* = −17→17
Absorption correction: multi-scan (*SADABS*; Bruker, 2014)	*k* = −6→6
*T*_min_ = 0.84, *T*_max_ = 0.90	*l* = −20→20
11488 measured reflections	

### Refinement

**Table:**

Refinement on *F*^2^	Secondary atom site location: difference Fourier map
Least-squares matrix: full	Hydrogen site location: mixed
*R*[*F*^2^ > 2σ(*F*^2^)] = 0.023	H atoms treated by a mixture of independent and constrained refinement
*wR*(*F*^2^) = 0.052	*w* = 1/[σ^2^(*F*_o_^2^) + (0.0137*P*)^2^ + 1.951*P*] where *P* = (*F*_o_^2^ + 2*F*_c_^2^)/3
*S* = 1.03	(Δ/σ)_max_ = 0.001
2420 reflections	Δρ_max_ = 0.29 e Å^−^^3^
207 parameters	Δρ_min_ = −0.21 e Å^−^^3^
36 restraints	Absolute structure: Flack *x* determined using 959 quotients [(*I*^+^)–(*I*^–^)]/[(*I*^+^)+(*I*^–^)] (Parsons *et al.*, 2013)
Primary atom site location: structure-invariant direct methods	Absolute structure parameter: −0.02 (2)

### Special details

**Table:**

Experimental. *M*.p. 365–366 K (not corrected); [*α*]^20^_D_ + 33.8 (*c* 0.32, CHCl_3_); IR (KBr): 3450, 3393, 3371, 3295, 3020, 2947, 2921, 2873, 1715, 1656, 1613, 1579, 1438, 1376, 1336, 1313, 1251, 1220, 1121, 1098, 1080, 1037, 1003, 977, 933, 909, 826, 803, 740, 585 cm^−1^; ^1^H NMR (500 MHz, CDCl_3_): *δ* (p.p.m.) 7.12 (dq, *J* = 7.5, 1.4 Hz, 1H; H13), 5.49 (t, *J* = 7.5 Hz, 1H; H8), 4.74 (dt, *J* = 7.5, 2.3 Hz, 1H; H9), 4.63 (bs, 1H; H12), 3.90 (dd, *J* = 12.9, 2.3 Hz, 1H; H11A), 3.77 (s, 3H; H19A–F), 3.57 (bd, *J* = 12.9 Hz, 1H; H11B), 3.06 (bs, 2H; H6), 1.90 (d, *J* = 1.4 Hz, 3H; H15A–F); ^13^C NMR (125 MHz, CDCl_3_): *δ* (p.p.m.) 167.6 (C), 135.6 (CH), 131.7 (C), 115.3 (C), 103.8 (C), 83.6 (CH), 79.8 (CH), 62.1 (CH_2_), 52.4 (CH_3_), 13.5 (CH_3_)
Geometry. All e.s.d.'s (except the e.s.d. in the dihedral angle between two l.s. planes) are estimated using the full covariance matrix. The cell e.s.d.'s are taken into account individually in the estimation of e.s.d.'s in distances, angles and torsion angles; correlations between e.s.d.'s in cell parameters are only used when they are defined by crystal symmetry. An approximate (isotropic) treatment of cell e.s.d.'s is used for estimating e.s.d.'s involving l.s. planes.
Refinement. Refinement of **F**^2^ against ALL reflections. The weighted **R**-factor *wR* and goodness of fit **S** are based on **F**^2^, conventional **R**-factors **R** are based on **F**, with **F** set to zero for negative **F**^2^. The threshold expression of **F**^2^ > 2*σ*(**F**^2^) is used only for calculating **R**-factors(gt) *etc.* and is not relevant to the choice of reflections for refinement. **R**-factors based on **F**^2^ are statistically about twice as large as those based on **F**, and **R**-factors based on ALL data will be even larger. Problematic three reflections (1 − 5 10, −13 0 15 and −12 0 16) with |**I**_obs_–**I**_calc_|/*σ*W(**I**) greater than 10 have been omitted in the final refinement.

### Fractional atomic coordinates and isotropic or equivalent isotropic displacement parameters (Å^2^)

**Table:**

	*x*	*y*	*z*	*U*_iso_*/*U*_eq_	Occ. (<1)
Cl1	0.49607 (5)	0.41064 (14)	0.10141 (4)	0.02169 (18)	
Cl2	0.69020 (5)	0.44496 (13)	0.18886 (5)	0.02292 (19)	
Cl3	0.57725 (5)	0.87077 (13)	0.15202 (5)	0.0279 (2)	
C4	0.5774 (2)	0.5655 (5)	0.17849 (19)	0.0165 (7)	
C5	0.5517 (2)	0.5446 (6)	0.25983 (19)	0.0192 (7)	
N6	0.4607 (2)	0.6381 (8)	0.25371 (19)	0.0432 (10)	
H6A	0.453 (6)	0.657 (12)	0.301 (2)	0.052*	0.5
H6B	0.453 (4)	0.781 (5)	0.236 (3)	0.052*	0.75
H6C	0.433 (2)	0.523 (6)	0.230 (3)	0.052*	0.75
O7	0.61956 (15)	0.6675 (4)	0.31855 (13)	0.0202 (5)	
C8	0.6707 (2)	0.5008 (6)	0.37781 (19)	0.0190 (7)	
H8	0.7322	0.4656	0.3673	0.023*	
C9	0.6103 (2)	0.2743 (6)	0.36247 (19)	0.0206 (7)	
H9	0.6519	0.1318	0.3659	0.025*	
O10	0.55577 (18)	0.3032 (4)	0.28050 (13)	0.0267 (6)	
C11	0.5478 (2)	0.2341 (6)	0.4164 (2)	0.0227 (7)	
H11A	0.5078	0.0928	0.3971	0.027*	
H11B	0.5867	0.1992	0.4715	0.027*	
O12	0.49006 (15)	0.4361 (5)	0.41859 (12)	0.0263 (5)	
H12A	0.496 (4)	0.488 (9)	0.4653 (18)	0.039*	0.5
H12B	0.465 (5)	0.491 (14)	0.374 (2)	0.039*	0.5
C13	0.6872 (2)	0.6143 (6)	0.45876 (19)	0.0230 (7)	
H13	0.647	0.7423	0.4636	0.028*	
C14	0.7528 (2)	0.5524 (7)	0.5242 (2)	0.0294 (9)	
C15A	0.818 (3)	0.343 (6)	0.5238 (16)	0.044 (3)	0.482 (5)
H15A	0.8202	0.311	0.4686	0.066*	0.482 (5)
H15B	0.7958	0.2007	0.5458	0.066*	0.482 (5)
H15C	0.8813	0.3833	0.5568	0.066*	0.482 (5)
C16A	0.767 (4)	0.651 (8)	0.6034 (12)	0.033 (4)	0.482 (5)
O17A	0.8138 (4)	0.5539 (12)	0.6617 (3)	0.0250 (10)	0.482 (5)
O18A	0.7178 (8)	0.851 (4)	0.5940 (10)	0.028 (2)	0.482 (5)
C19A	0.7246 (9)	0.960 (3)	0.6710 (10)	0.032 (2)	0.482 (5)
H19A	0.6983	0.8512	0.7037	0.047*	0.482 (5)
H19B	0.6899	1.1108	0.6631	0.047*	0.482 (5)
H19C	0.7904	0.9908	0.6985	0.047*	0.482 (5)
C15B	0.825 (2)	0.365 (6)	0.5320 (16)	0.044 (3)	0.518 (5)
H15D	0.8045	0.2507	0.4876	0.066*	0.518 (5)
H15E	0.8331	0.2813	0.5832	0.066*	0.518 (5)
H15F	0.8837	0.439	0.5302	0.066*	0.518 (5)
C16B	0.757 (3)	0.706 (7)	0.5989 (12)	0.033 (4)	0.518 (5)
O17B	0.8182 (4)	0.6902 (11)	0.6645 (3)	0.0250 (10)	0.518 (5)
O18B	0.6889 (7)	0.875 (3)	0.5956 (10)	0.028 (2)	0.518 (5)
C19B	0.6959 (9)	1.028 (2)	0.6652 (9)	0.032 (2)	0.518 (5)
H19D	0.6942	0.9286	0.7115	0.047*	0.518 (5)
H19E	0.6433	1.1402	0.6535	0.047*	0.518 (5)
H19F	0.7547	1.1171	0.6773	0.047*	0.518 (5)

### Atomic displacement parameters (Å^2^)

**Table:**

	*U*^11^	*U*^22^	*U*^33^	*U*^12^	*U*^13^	*U*^23^
Cl1	0.0302 (4)	0.0189 (4)	0.0146 (3)	−0.0018 (4)	0.0037 (3)	−0.0028 (3)
Cl2	0.0207 (4)	0.0251 (5)	0.0273 (4)	0.0083 (3)	0.0141 (3)	0.0056 (4)
Cl3	0.0237 (4)	0.0137 (4)	0.0420 (5)	0.0004 (3)	0.0018 (4)	0.0047 (4)
C4	0.0155 (16)	0.0148 (16)	0.0214 (16)	0.0008 (12)	0.0086 (13)	−0.0014 (13)
C5	0.0131 (17)	0.0287 (18)	0.0165 (17)	−0.0020 (13)	0.0051 (13)	−0.0073 (14)
N6	0.0131 (15)	0.089 (3)	0.0263 (18)	0.0115 (18)	0.0024 (13)	−0.0247 (19)
O7	0.0208 (12)	0.0169 (12)	0.0172 (11)	0.0036 (9)	−0.0047 (9)	−0.0012 (9)
C8	0.0137 (15)	0.0210 (18)	0.0223 (17)	0.0045 (13)	0.0048 (13)	0.0037 (13)
C9	0.0240 (18)	0.0197 (17)	0.0206 (16)	0.0036 (14)	0.0104 (14)	0.0002 (14)
O10	0.0369 (15)	0.0285 (14)	0.0170 (12)	−0.0178 (11)	0.0112 (11)	−0.0035 (10)
C11	0.0295 (19)	0.0227 (19)	0.0208 (17)	−0.0012 (15)	0.0150 (15)	−0.0007 (14)
O12	0.0254 (12)	0.0362 (15)	0.0209 (11)	0.0051 (12)	0.0123 (10)	−0.0023 (12)
C13	0.0171 (16)	0.0258 (19)	0.0244 (18)	−0.0025 (14)	0.0027 (14)	0.0030 (15)
C14	0.0161 (18)	0.042 (2)	0.0273 (19)	−0.0112 (15)	0.0012 (15)	0.0131 (17)
C15A	0.021 (4)	0.066 (5)	0.039 (4)	0.007 (4)	0.000 (4)	0.030 (4)
C16A	0.014 (8)	0.065 (16)	0.018 (3)	−0.015 (6)	0.000 (2)	0.014 (5)
O17A	0.0230 (16)	0.033 (3)	0.0179 (15)	−0.003 (3)	0.0029 (13)	0.002 (3)
O18A	0.022 (6)	0.039 (4)	0.0231 (14)	−0.011 (5)	0.006 (4)	−0.0089 (19)
C19A	0.031 (7)	0.034 (7)	0.031 (3)	−0.005 (4)	0.012 (5)	−0.017 (4)
C15B	0.021 (4)	0.066 (5)	0.039 (4)	0.007 (4)	0.000 (4)	0.030 (4)
C16B	0.014 (8)	0.065 (16)	0.018 (3)	−0.015 (6)	0.000 (2)	0.014 (5)
O17B	0.0230 (16)	0.033 (3)	0.0179 (15)	−0.003 (3)	0.0029 (13)	0.002 (3)
O18B	0.022 (6)	0.039 (4)	0.0231 (14)	−0.011 (5)	0.006 (4)	−0.0089 (19)
C19B	0.031 (7)	0.034 (7)	0.031 (3)	−0.005 (4)	0.012 (5)	−0.017 (4)

### Geometric parameters (Å, º)

**Table:**

Cl1—C4	1.763 (3)	C13—H13	0.95
Cl2—C4	1.772 (3)	C14—C16A	1.439 (17)
Cl3—C4	1.765 (3)	C14—C15B	1.475 (17)
C4—C5	1.560 (4)	C14—C15A	1.524 (18)
C5—O10	1.392 (4)	C14—C16B	1.538 (16)
C5—O7	1.401 (4)	C15A—H15A	0.98
C5—N6	1.428 (5)	C15A—H15B	0.98
N6—H6A	0.87 (3)	C15A—H15C	0.98
N6—H6B	0.85 (2)	C16A—O17A	1.19 (2)
N6—H6C	0.81 (2)	C16A—O18A	1.318 (19)
O7—C8	1.439 (4)	O18A—C19A	1.442 (17)
C8—C13	1.495 (4)	C19A—H19A	0.98
C8—C9	1.532 (4)	C19A—H19B	0.98
C8—H8	1.0	C19A—H19C	0.98
C9—O10	1.437 (4)	C15B—H15D	0.98
C9—C11	1.502 (5)	C15B—H15E	0.98
C9—H9	1.0	C15B—H15F	0.98
C11—O12	1.425 (4)	C16B—O17B	1.252 (19)
C11—H11A	0.99	C16B—O18B	1.370 (18)
C11—H11B	0.99	O18B—C19B	1.455 (16)
O12—H12A	0.84 (3)	C19B—H19D	0.98
O12—H12B	0.82 (3)	C19B—H19E	0.98
C13—C14	1.323 (5)	C19B—H19F	0.98
			
C5—C4—Cl1	111.0 (2)	C14—C13—C8	125.8 (3)
C5—C4—Cl3	108.9 (2)	C14—C13—H13	117.1
Cl1—C4—Cl3	109.02 (17)	C8—C13—H13	117.1
C5—C4—Cl2	109.8 (2)	C13—C14—C16A	126.6 (9)
Cl1—C4—Cl2	109.01 (16)	C13—C14—C15B	127.6 (11)
Cl3—C4—Cl2	109.09 (16)	C13—C14—C15A	121.4 (11)
O10—C5—O7	108.4 (3)	C16A—C14—C15A	111.8 (12)
O10—C5—N6	110.3 (3)	C13—C14—C16B	115.0 (7)
O7—C5—N6	110.8 (3)	C15B—C14—C16B	117.3 (13)
O10—C5—C4	107.5 (3)	C14—C15A—H15A	109.5
O7—C5—C4	108.2 (3)	C14—C15A—H15B	109.5
N6—C5—C4	111.5 (3)	H15A—C15A—H15B	109.5
C5—N6—H6A	110 (5)	C14—C15A—H15C	109.5
C5—N6—H6B	113 (4)	H15A—C15A—H15C	109.5
H6A—N6—H6B	101 (5)	H15B—C15A—H15C	109.5
C5—N6—H6C	95 (2)	O17A—C16A—O18A	132.0 (16)
H6A—N6—H6C	113 (5)	O17A—C16A—C14	122.1 (15)
H6B—N6—H6C	124 (5)	O18A—C16A—C14	106.0 (14)
C5—O7—C8	109.6 (2)	C16A—O18A—C19A	110.2 (15)
O7—C8—C13	108.2 (2)	O18A—C19A—H19A	109.5
O7—C8—C9	103.9 (2)	O18A—C19A—H19B	109.5
C13—C8—C9	116.8 (3)	H19A—C19A—H19B	109.5
O7—C8—H8	109.2	O18A—C19A—H19C	109.5
C13—C8—H8	109.2	H19A—C19A—H19C	109.5
C9—C8—H8	109.2	H19B—C19A—H19C	109.5
O10—C9—C11	110.5 (3)	C14—C15B—H15D	109.5
O10—C9—C8	103.0 (2)	C14—C15B—H15E	109.5
C11—C9—C8	116.7 (3)	H15D—C15B—H15E	109.5
O10—C9—H9	108.8	C14—C15B—H15F	109.5
C11—C9—H9	108.8	H15D—C15B—H15F	109.5
C8—C9—H9	108.8	H15E—C15B—H15F	109.5
C5—O10—C9	109.6 (2)	O17B—C16B—O18B	115.7 (13)
O12—C11—C9	112.2 (3)	O17B—C16B—C14	124.9 (15)
O12—C11—H11A	109.2	O18B—C16B—C14	119.4 (15)
C9—C11—H11A	109.2	C16B—O18B—C19B	119.0 (13)
O12—C11—H11B	109.2	O18B—C19B—H19D	109.5
C9—C11—H11B	109.2	O18B—C19B—H19E	109.5
H11A—C11—H11B	107.9	H19D—C19B—H19E	109.5
C11—O12—H12A	113 (2)	O18B—C19B—H19F	109.5
C11—O12—H12B	113 (6)	H19D—C19B—H19F	109.5
H12A—O12—H12B	133 (6)	H19E—C19B—H19F	109.5
			
Cl1—C4—C5—O10	62.2 (3)	C8—C9—O10—C5	−22.5 (3)
Cl3—C4—C5—O10	−177.8 (2)	O10—C9—C11—O12	−64.2 (3)
Cl2—C4—C5—O10	−58.5 (3)	C8—C9—C11—O12	52.9 (4)
Cl1—C4—C5—O7	179.0 (2)	O7—C8—C13—C14	−158.9 (3)
Cl3—C4—C5—O7	−61.0 (3)	C9—C8—C13—C14	84.4 (4)
Cl2—C4—C5—O7	58.4 (3)	C8—C13—C14—C16A	−177 (4)
Cl1—C4—C5—N6	−58.9 (4)	C8—C13—C14—C15B	2 (2)
Cl3—C4—C5—N6	61.2 (3)	C8—C13—C14—C15A	−2 (2)
Cl2—C4—C5—N6	−179.5 (3)	C8—C13—C14—C16B	178 (3)
O10—C5—O7—C8	1.5 (4)	C13—C14—C16A—O17A	164 (4)
N6—C5—O7—C8	122.7 (3)	C15A—C14—C16A—O17A	−11 (7)
C4—C5—O7—C8	−114.7 (3)	C13—C14—C16A—O18A	−15 (6)
C5—O7—C8—C13	−139.8 (3)	C15A—C14—C16A—O18A	170 (4)
C5—O7—C8—C9	−15.0 (3)	O17A—C16A—O18A—C19A	−1 (8)
O7—C8—C9—O10	22.3 (3)	C14—C16A—O18A—C19A	178 (3)
C13—C8—C9—O10	141.3 (3)	C13—C14—C16B—O17B	−174 (4)
O7—C8—C9—C11	−98.9 (3)	C15B—C14—C16B—O17B	3 (7)
C13—C8—C9—C11	20.1 (4)	C13—C14—C16B—O18B	6 (6)
O7—C5—O10—C9	14.0 (4)	C15B—C14—C16B—O18B	−177 (4)
N6—C5—O10—C9	−107.5 (3)	O17B—C16B—O18B—C19B	3 (6)
C4—C5—O10—C9	130.7 (2)	C14—C16B—O18B—C19B	−177 (3)
C11—C9—O10—C5	102.8 (3)		

### Hydrogen-bond geometry (Å, º)

**Table:**

*D*—H···*A*	*D*—H	H···*A*	*D*···*A*	*D*—H···*A*
O12—H12*B*···N6	0.82 (3)	2.22 (4)	2.986 (4)	155 (7)
N6—H6*A*···O12	0.87 (3)	2.31 (5)	2.986 (4)	135 (6)
O12—H12*A*···O12^i^	0.84 (3)	1.98 (4)	2.750 (4)	151 (5)
N6—H6*C*···O17*B*^ii^	0.81 (2)	2.57 (2)	3.369 (7)	169 (3)
C19*A*—H19*C*···N6^iii^	0.98	2.59	3.555 (15)	167
N6—H6*B*···O17*A*^iv^	0.85 (2)	2.61 (5)	3.286 (7)	137 (5)
C19*B*—H19*E*···O12^v^	0.98	2.62	3.573 (11)	164

Symmetry codes: (i) −*x*+1, *y*, −*z*+1; (ii) *x*−1/2, *y*−1/2, *z*−1/2; (iii) *x*+1/2, *y*+1/2, *z*+1/2; (iv) *x*−1/2, *y*+1/2, *z*−1/2; (v) −*x*+1, *y*+1, −*z*+1.
